# Multiple sarcomatoid carcinomas in the small intestine with perforation: A case report and literature review

**DOI:** 10.1097/MD.0000000000038147

**Published:** 2024-05-10

**Authors:** Xujie Wang, Huan Zhang, Long Li, Jixin Fu, Xinjian Wang

**Affiliations:** a Department of Gastrointestinal Surgery, Weihai Central Hospital, Qingdao University, Weihai, China; b Department of Colorectal Disease, Shanghai Tenth People’s Hospital, Tongji University School of Medicine, Shanghai, China.

**Keywords:** early detection, early treatment, enhanced examination, sarcomatoid carcinoma of the small intestine

## Abstract

**Rationale:**

Sarcomatoid carcinoma of the small intestine is an exceedingly rare and aggressive malignancy, often diagnosed at advanced stages with a poor prognosis. This study documents a detailed case of sarcomatoid carcinoma of the small intestine, highlighting the diagnostic challenges and treatment approaches, underscored by a comprehensive review of related literature. Given the rarity of this condition, our report aims to enrich the existing diagnostic and treatment frameworks for this malignancy, emphasizing the necessity for early detection and intervention strategies. By presenting this case in conjunction with a literature review, we seek to shed light on the elusive nature of sarcomatoid carcinoma in the small intestine and propose avenues for improving patient outcomes.

**Patient concerns:**

Case presentation A 61-year-old male patient initially presented with recurrent abdominal pain and gastrointestinal symptoms. Initial abdominal computed tomography (CT) scans and gastrointestinal endoscopy revealed only inflammatory and hyperplastic changes in the duodenum and jejunum, with a diagnosis of intestinal obstruction. Two years later, due to gastrointestinal perforation, the patient was hospitalized again.

**Diagnoses:**

CT scans and other examinations revealed small intestinal lesions. Four small intestinal lesions were surgically removed, and pathology and immunohistochemistry confirmed sarcomatoid carcinoma of the small intestine. A short time later, enhanced CT scans revealed metastatic lesions in the hepatic portal and adrenal glands.

**Interventions:**

After surgery, the gastrointestinal function gradually recovered, and the patient was discharged from the hospital on a semiliquid diet. No further treatment such as radiotherapy or chemotherapy was administered postoperatively.

**Outcomes:**

Five months after the surgery, the patient died due to brain metastasis.

**Lessons:**

The study outcomes reveal the aggressive nature of sarcomatoid carcinoma of the small intestine, characterized by rapid progression and poor prognosis despite surgical interventions. The patient condition rapidly deteriorated, leading to metastasis and death within 5 months postsurgery. These findings underscore the critical need for early detection and possibly innovative treatment approaches to improve survival rates. This case also highlights the potential for gastrointestinal sarcomatoid carcinoma to metastasize to distant organs, including the brain, suggesting a propensity for hematogenous spread.

## 1. Introduction

The incidence of primary malignant tumors in the small intestine is low, accounting for 1% to 3% of all gastrointestinal malignancies. The most common pathological types are adenocarcinoma, neuroendocrine tumors, lymphoma, and gastrointestinal stromal tumors.^[1,2]^ Sarcomatoid carcinoma is rare. Morphologically resembling sarcomas, sarcomatoid carcinoma is currently believed to be a malignant tumor of epithelial origin^.[3,4]^ It can occur in various organ systems throughout the body but is less common in the gastrointestinal tract, especially in the small intestine, where it is extremely rare. Sarcomatoid carcinoma of the small intestine is often in the middle and late stages when discovered. Diagnosis relies on pathology and immunohistochemical examination, and surgery is the main treatment method, with a poor prognosis.^[5]^ Our hospital has treated 1 case, with more than 2 years of diagnosis and treatment. By reviewing the patient diagnosis and treatment experience and literature review, we hope to provide a reference for the diagnosis and treatment decision-making of sarcomatoid carcinoma of the small intestine.

## 2. Case presentation

**Figure 1.**
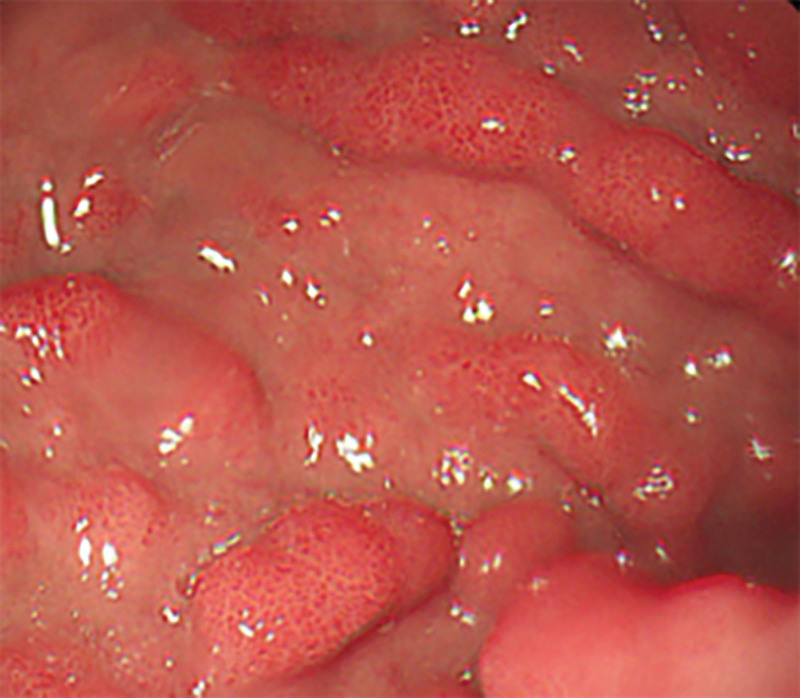
Endoscopic view showing mucosal congestion, edema, scattered mucosal elevations with cobblestone-like changes, and localized ulceration.

On May 9, 2020, a 61-year-old male patient was admitted to the hospital with a complaint of “abdominal pain and bloating for 1 week, aggravated for 1 day.” Physical examination revealed a soft abdomen, absence of peristalsis, diffuse tenderness in the entire abdomen with the upper abdomen being more pronounced, no rebound tenderness, and no palpable masses. Routine blood tests showed a slightly low hemoglobin level of 126 g/L. The tumor marker carcinoembryonic antigen was slightly elevated at 5.3 ng/mL, while carbohydrate antigen 19-9 was within the normal range at 10.8 U/mL. An abdominal computed tomography (CT) scan suggested possible intestinal obstruction, but no space-occupying lesions were detected. Gastroscopy revealed congested mucosa in the gastric body and localized erosion near the lesser curvature side of the gastric antrum, with a tough texture. Biopsy samples were taken from these areas. The duodenal bulb and descending part showed congested mucosa, scattered mucosal elevations, cobblestone-like changes, localized erosion, and a tough texture. Biopsy samples were also obtained from these areas (Fig. 1). Both biopsy samples showed chronic inflammation of the mucosa, with some evident glandular hyperplasia, but no tumor changes were found. The colonoscopy did not reveal any obvious masses in the cecum, ascending colon, transverse colon, descending colon, or sigmoid colon. However, a polyp measuring approximately 0.8 cm in diameter was found approximately 5 cm from the anal verge. After supportive treatments such as fasting, acid suppression, and fluid resuscitation, the patient symptoms of intestinal obstruction improved, and he was discharged on May 15, 2020.

**Figure 2.**
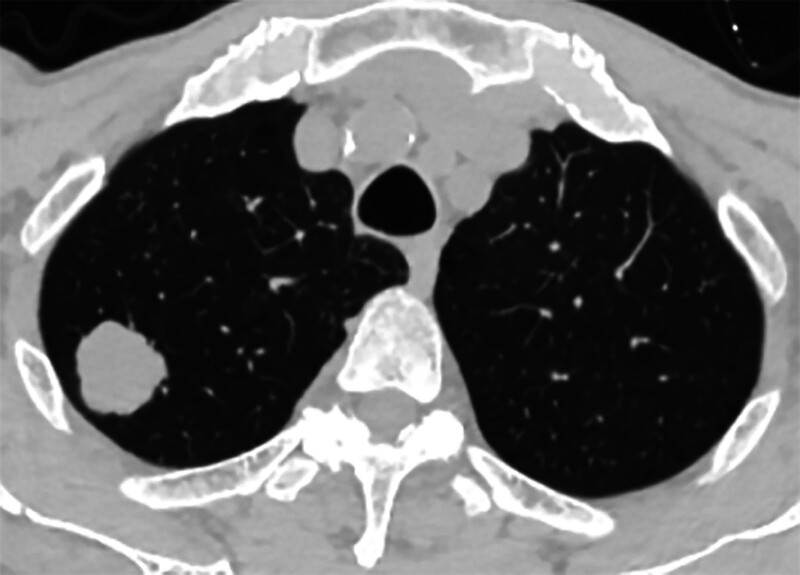
Right upper lobe lung mass.

**Figure 3.**
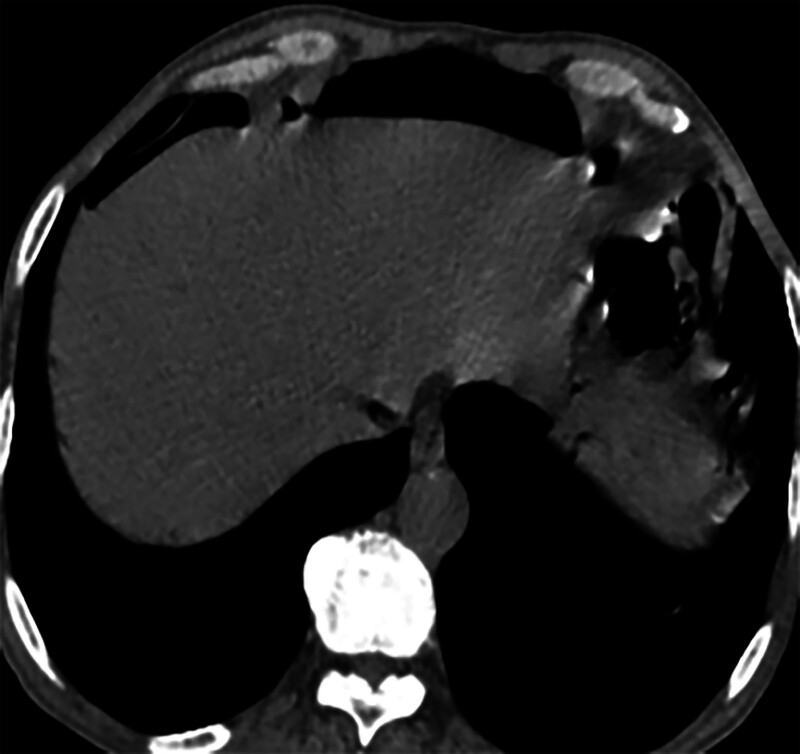
Gastrointestinal perforation.

**Figure 4.**
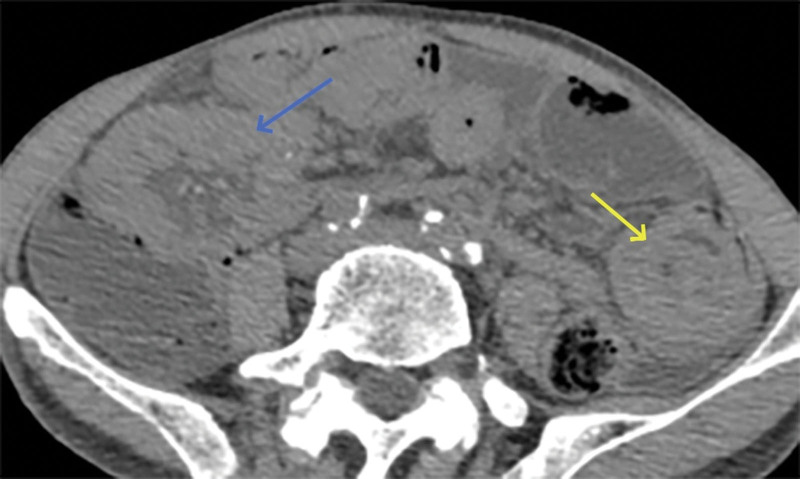
Localized thickening of the small bowel wall (blue arrow) and sign of intestinal stacking (yellow arrow).

**Figure 5.**
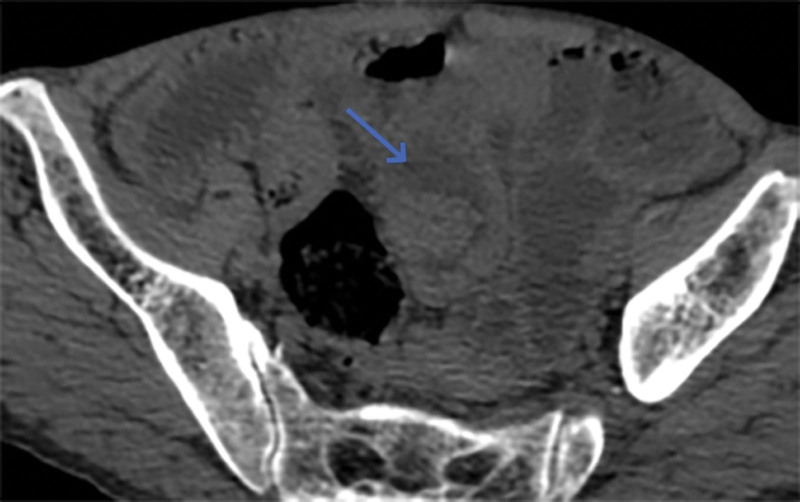
Localized thickening of the small bowel wall (2).

**Figure 6.**
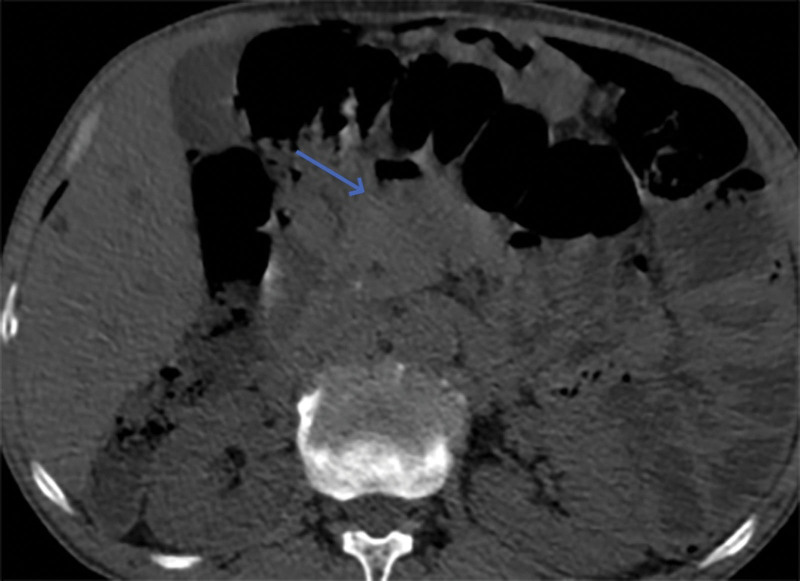
Localized thickening of the small bowel wall (3).

**Figure 7.**
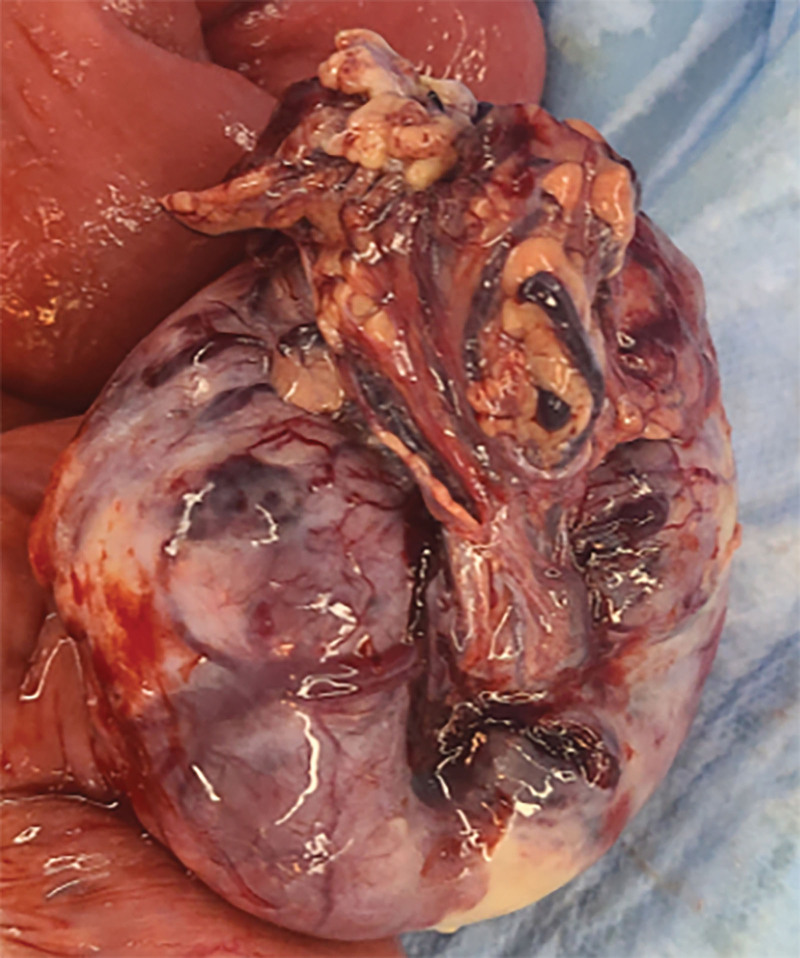
Intraoperative findings of small bowel tumors (1).

**Figure 8.**
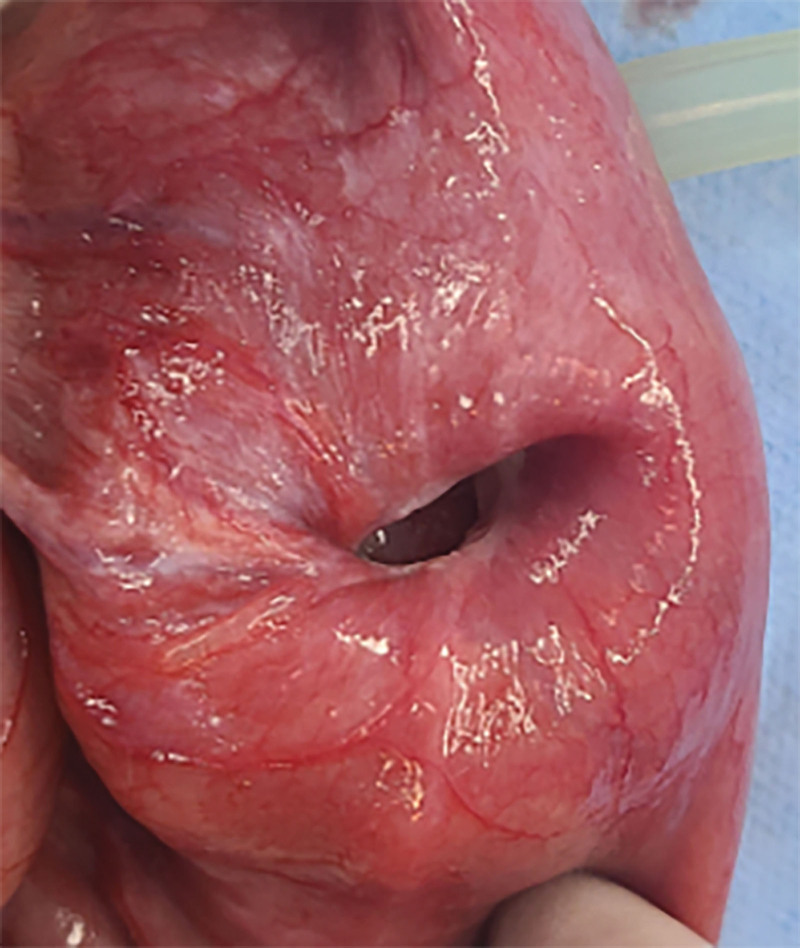
Intraoperative findings of small bowel tumors (2).

**Figure 9.**
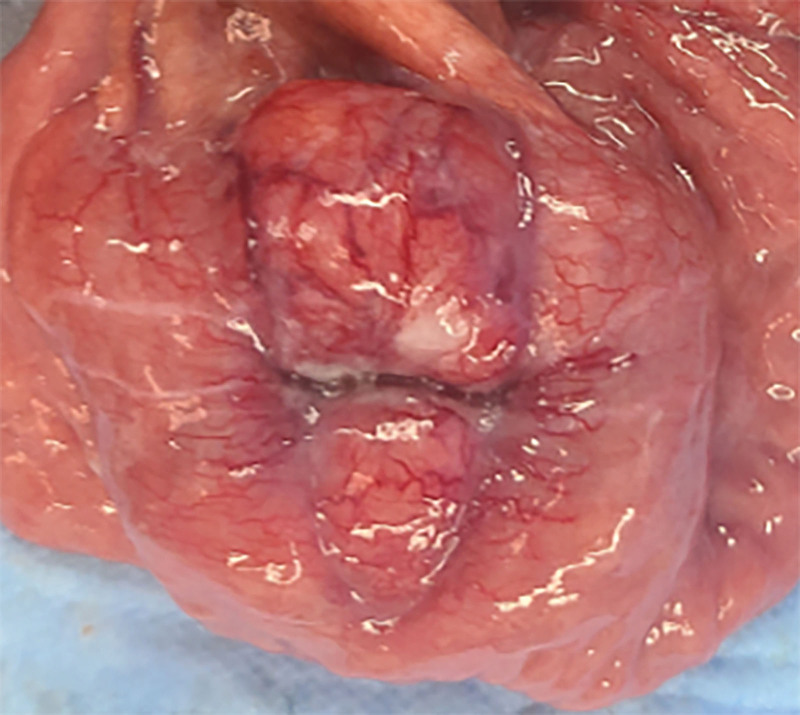
Intraoperative findings of small bowel tumors (3).

**Figure 10.**
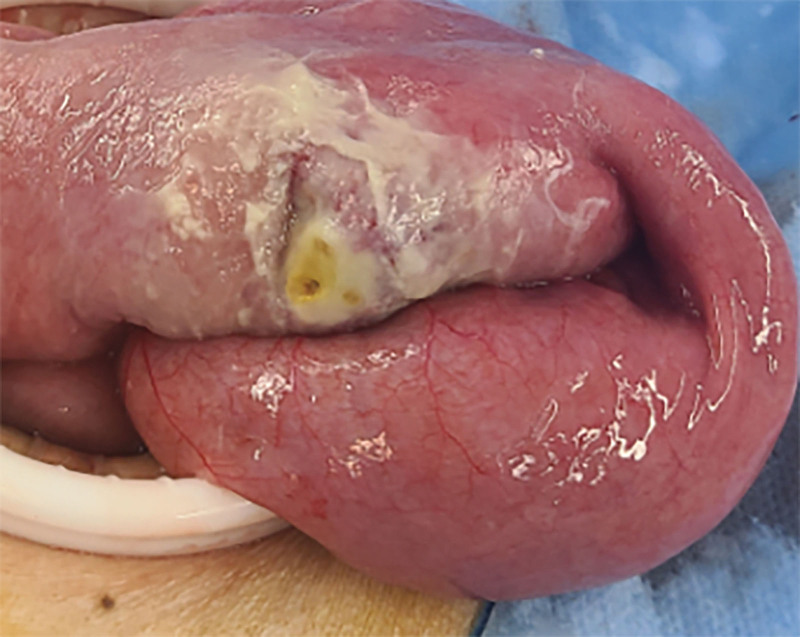
Intraoperative findings of small bowel tumors (4).

**Figure 11.**
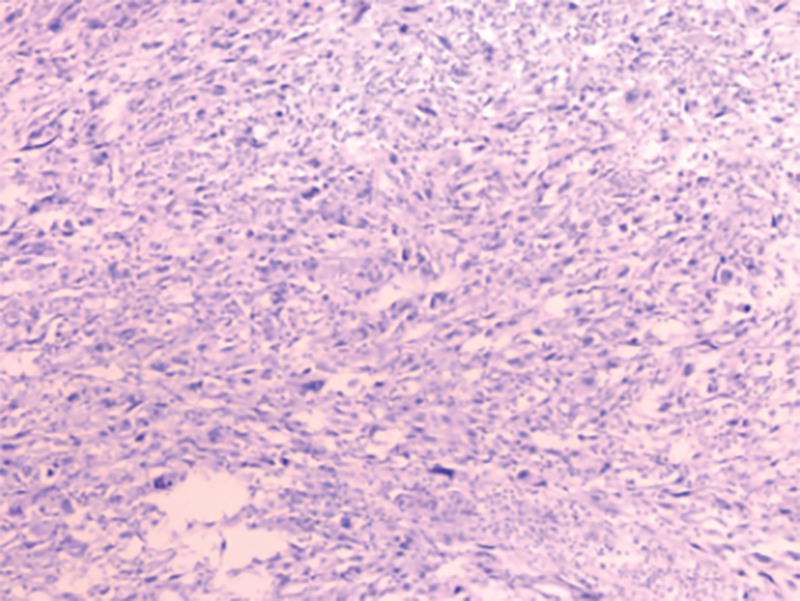
Pathological images of surgical specimens (1).

**Figure 12.**
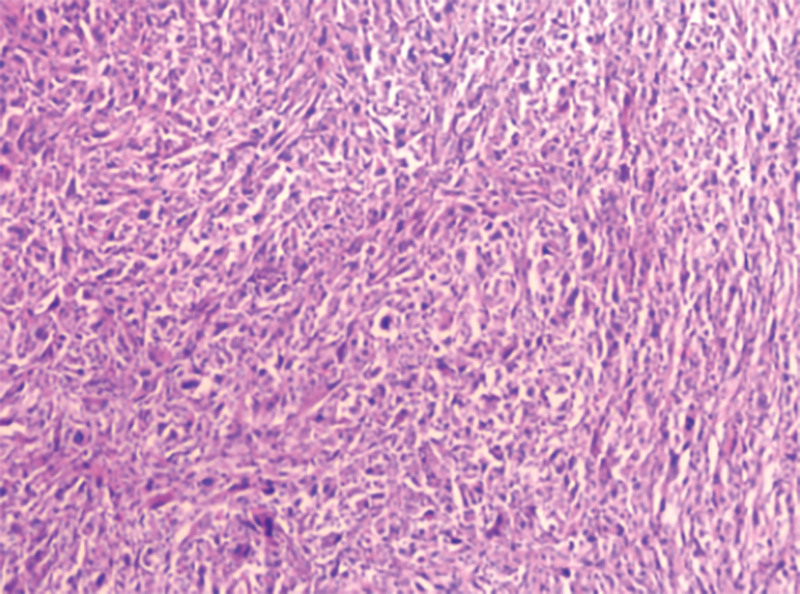
Pathological images of surgical specimens (2).

**Figure 13.**
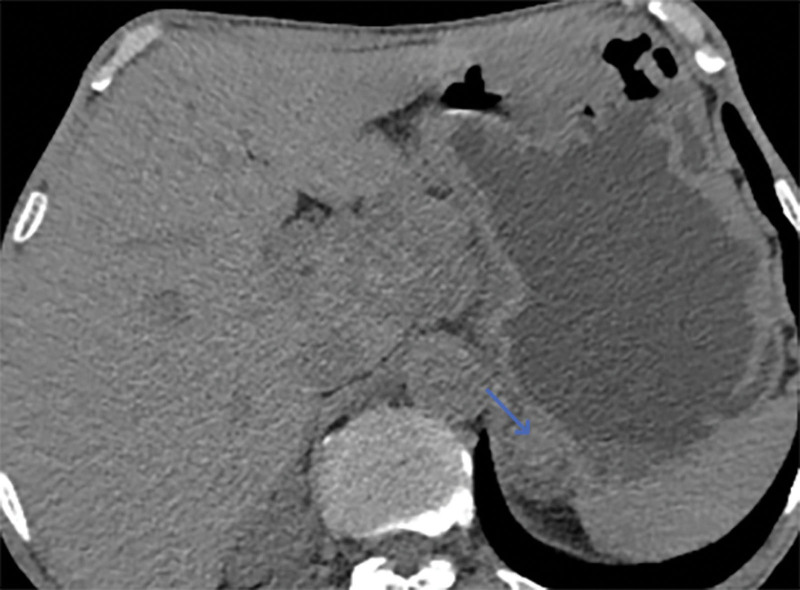
Blue arrow points to the adrenal mass in the left kidney.

**Figure 14.**
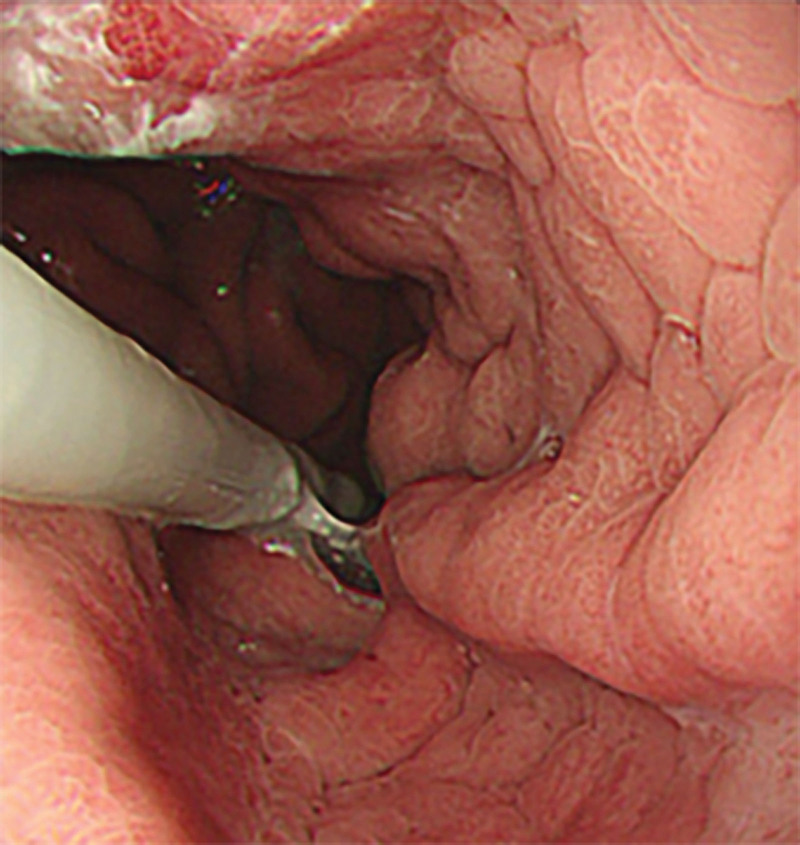
Gastroscopic view taken 24 d after surgery, showing diffuse congested and edematous gastric mucosa, with prominent folds and swelling.

**Figure 15.**
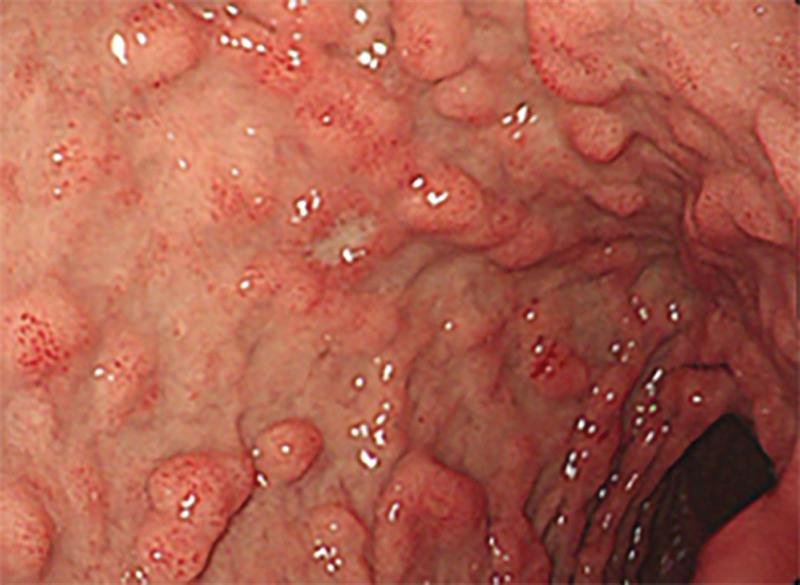
Gastroscopic view taken 24 d after surgery, revealing congested and edematous duodenal mucosa with nodular enlargement, along with multiple small ulcer formations and a white coating on the base.

**Figure 16.**
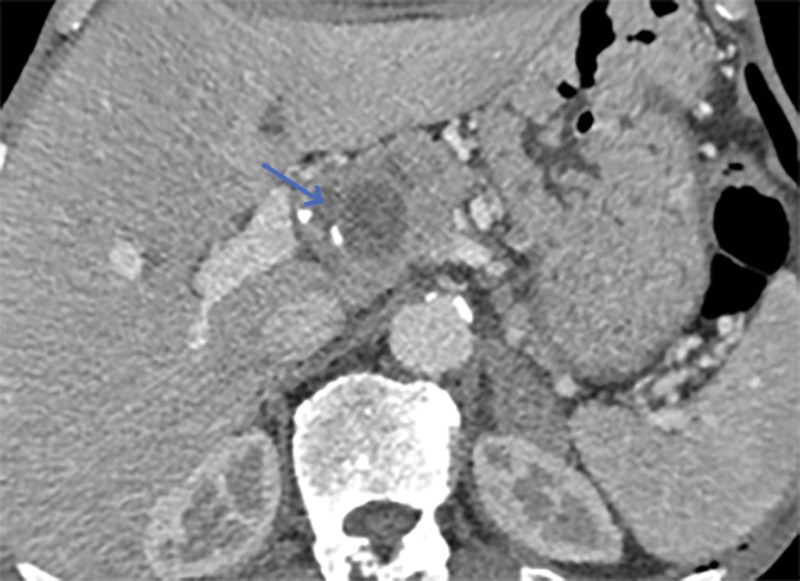
Blue arrow points to a mass in the porta hepatis.

However, on September 28, 2022, the patient was readmitted due to “sudden abdominal pain for 8 hours and fever for 30 minutes.” The patient hemoglobin level was 99 g/L, indicating mild anemia. Carcinoembryonic antigen and carbohydrate antigen19-9 levels were within the normal range. A chest CT scan showed a possible tumor-like lesion in the right upper lobe of the lung (Fig. 2), along with multiple nodules in both lungs. An abdominal CT scan suggested gastrointestinal perforation (Fig. 3), intraabdominal fluid accumulation, localized intestinal wall thickening, and possible intestinal intussusception (Figs. 4–6). Emergency surgery was performed, and exploration revealed the presence of 4 tumors in the small intestine (Figs. 7–10). These tumors invaded the serosa and measured 7 cm × 4.5 cm × 3 cm (30 cm from the Treitz ligament), 5 cm × 4 cm × 2.5 cm (60 cm from the Treitz ligament), 5.5 cm × 2.7 cm (150 cm from the Treitz ligament), and 8 cm × 5 cm × 3.5 cm (170 cm from the Treitz ligament). Among them, the tumor located 170 cm from the Treitz ligament exhibited central necrosis and perforation. The tumors and their corresponding mesenteries were completely resected. Pathological examination revealed (Figs. 11 and 12) that the majority of tumor cells were spindle-shaped with significant cellular atypia and visible mitotic figures, accompanied by areas of necrosis. Based on immunohistochemistry, the diagnosis was undifferentiated sarcoma or sarcomatoid carcinoma of epithelial origin, with no lymph node metastasis. Immunohistochemical markers were positive for vimentin, CK (P), and CD34 (vascular) and negative for DOG1, CD117, SMA, S-100, CD20, desmin, CR, D2-40, WT-1, HMB45, MelanA, and Ki67 (70%+). Postoperatively, the patient received antimicrobial therapy and enteral and parenteral nutrition support. However, the recovery of gastrointestinal function was slow, and the patient experienced recurrent symptoms. Fourteen days after the surgery, a follow-up abdominal CT revealed a mass in the left adrenal gland (Fig. 13). Twenty-four days postsurgery, gastroscopy showed diffuse congested and edematous mucosa in the gastric body and antrum (Fig. 14). The duodenal bulb and descending part exhibited diffuse congested and edematous mucosa, some with nodular enlargement, multiple small ulcerations, and white coating on the base (Fig. 15). Biopsy samples were taken, and the histopathological examination showed chronic mucosal inflammation. At 28 days postsurgery, an abdominal enhanced CT scan revealed a mass in the hepatic hilum (Fig. 16). One month after the surgery, the patient gradually resumed a semiliquid diet. However, in February 2023, the patient developed hemiplegia, and upon examination at an external hospital, brain metastasis was discovered. Unfortunately, the patient passed away.

This work has been carried out in accordance with the Declaration of Helsinki (2000) of the World Medical Association. This study was reviewed by the ethics committee of Weihai Central Hospital (2023-35). All human subjects provided written informed consents with guarantees of confidentiality.

## 3. Discussion

This case report presents a challenging diagnostic and therapeutic scenario involving a patient who initially presented with nonspecific abdominal pain and bloating. The patient subsequently experienced a course of chronic inflammation, intestinal obstruction, and inflammation in the stomach and duodenum, ultimately leading to the diagnosis of multiple sarcomatoid carcinomas in the small intestine. In this case, the early symptoms and physical signs did not distinctly indicate small intestine sarcomatoid carcinoma but rather resembled more common gastrointestinal disorders such as gastritis, duodenitis, and intestinal obstruction. Moreover, blood tumor markers did not exhibit a significant elevation. However, upon the occurrence of recurrent abdominal pain, further imaging studies revealed the presence of tumors.

Sarcomatoid carcinoma of the small intestine is rare and was first reported by Dikman and Toker in 1973. Its occurrence may be related to prolonged regional inflammation.^[6]^ According to statistics,^[7,8]^ there have been just over 30 reported cases to date, predominantly affecting middle-aged and elderly males, with a higher incidence in men than in women, and most cases are solitary tumors, with a higher occurrence in the jejunum than in the ileum. In this case, the patient was an elderly male with multiple lesions, and the tumors were mostly located in the jejunum, which is in line with the statistical data.

The clinical manifestations of small intestine sarcomatoid carcinoma lack tumor specificity and are mainly related to the site of occurrence. Common presentations include abdominal pain, intestinal obstruction, nausea, vomiting, abdominal masses, weight loss, gastrointestinal bleeding, and anemia, with anemia and abdominal pain being more frequently observed.^[9]^ In this patient’s first hospitalization, the symptoms included upper abdominal pain, nausea, vomiting, and intestinal obstruction, but there was no anemia. In the second hospitalization, the patient experienced gastrointestinal perforation, presenting with generalized abdominal pain, fever, and accompanying anemia, which is consistent with other reported cases.

When patients with sarcomatoid carcinoma of the small intestine show symptoms, CT, MR, ultrasound, gastrointestinal angiography, and endoscopy may reveal tumor lesions; however, tumor markers usually do not show specific elevations.^[10]^ CT findings commonly include circumferential thickening of the intestinal wall or intraluminal rounded soft tissue masses, with moderate to heterogeneous enhancement after contrast administration. Secondary signs may include intestinal obstruction, intussusception, small bowel dilatation, and intestinal fistula formation.^[11]^ Small intestine sarcomatoid carcinoma tends to progress rapidly, and at the time of diagnosis, the tumor lesions are often large and prone to metastasis.^[12]^ In this case, during the initial presentation, a CT scan did not detect any lesions, but gastroscopy revealed congested, edematous, and proliferative changes in the duodenum with a cobblestone-like appearance. Two years later, a repeat CT scan revealed tumor-like lesions in the small intestine with a diameter of 8 cm. The CT enhancement scan detected metastatic lesions in the adrenal gland and hepatic hilum, and there were also tumor lesions in the lungs. Gastroscopy showed further exacerbation of gastric mucosal swelling, with thickened folds and nodular enlargement in the duodenum. Less than 3 years later, the patient succumbed to brain metastasis. Tumor markers were only mildly elevated in 1 of the 2 tests and did not have significant diagnostic value for detecting the tumor. This case further confirms the high invasiveness and metastatic potential of small intestine sarcomatoid carcinoma. It also highlights that contrast-enhanced CT is more effective than nonenhanced CT in detecting lesions. Previous literature has not reported endoscopic findings of sarcomatoid carcinoma of the small intestine. The abnormal hyperplasia of the stomach and duodenum found in this patient’s endoscopy is highly likely to be sarcomatoid carcinoma. Biopsy of the mucosal surface cannot rule out sarcomatoid carcinoma and requires further endoscopic ultrasound, enhanced CT/MR, and other examinations to avoid missing the discovery of sarcomatoid carcinoma.

The diagnosis of small intestine sarcomatoid carcinoma relies on pathological examination and immunohistochemistry. Its characteristic features include polypoid or centrally ulcerated masses on gross examination, and microscopically, it exhibits sarcomatoid morphology with or without elements of epithelial differentiation. Immunohistochemical staining shows positive expression of both the epithelial marker CK (cytokeratin) and the mesenchymal marker vimentin.^[13]^ In this case, the pathological findings align with the aforementioned characteristics. Studies have shown that an increased Ki67 labeling index in immunohistochemistry often indicates a poorer prognosis.^[14]^ In this case, the patient had a Ki67 labeling index of 70%+, and the disease progressed rapidly from symptom onset to death in <3 years. During the course of the disease, multiple lesions rapidly appeared, and metastases occurred in various sites, which aligns well with this pattern.

According to domestic and foreign reports,^[8]^ the current radiotherapy and chemotherapy regimens are ineffective for small intestine sarcomatoid carcinoma. Radical resection with regional lymph node clearance can improve prognosis to some extent, but it is difficult to alter the overall outcome. Generally, the prognosis for small intestine sarcomatoid carcinoma is poor, with 70% of patients succumbing to the disease within 2 months to 3 years of follow-up^[15]^ and very few surviving beyond 5 years.^[16]^ In this case, the patient underwent resection of the small intestine lesion and perforation, which improved symptoms. However, the tumor continued to progress and metastasize, leading to death within 4 months after surgery, consistent with other reports. Notably, the occurrence of brain metastasis in this case underscores the aggressive nature of sarcomatoid carcinoma, presenting significant challenges for treatment and indicating the necessity for advanced research into potential preventative and therapeutic strategies. In recent years, there have been reports of specific expression of the programmed death ligand 1 (PD-L1) protein in small intestine sarcomatoid carcinoma, suggesting that PD-L1-targeted immunotherapy might be effective,^[9,17]^ and further verification and trials are warranted. Additionally, the genomic characteristics of small intestine sarcomatoid carcinoma have been identified in some studies,^[8]^ and further research may reveal its molecular pathogenesis and help discover new treatment approaches.

The primary limitations of this study stem from its nature as a single-case report, limiting the generalizability of our findings. Additionally, the rarity of sarcomatoid carcinoma in the small intestine poses significant challenges in accumulating a comprehensive dataset for analysis. Furthermore, our reliance on historical medical records and imaging may introduce retrospective biases. These limitations underscore the need for more extensive, multi-center studies to validate our observations and conclusions.

## 4. Conclusion

The incidence of tumors in the small intestine is low, and sarcomatoid carcinoma of the small intestine is exceedingly rare. Although the clinical manifestations are nonspecific, lesions are often detectable through enhanced CT/MR examinations. This case study demonstrates that, even when diagnosed at an advanced stage, surgical resection remains the primary method for removing identified lesions. However, current radiotherapy and chemotherapy regimens are ineffective, leading to poor prognosis for the tumor.^[12]^ Therefore, for patients with persistent abdominal pain, unexplained anemia, intestinal obstruction, weight loss, or suspected lesions of the small intestine, it is crucial to enhance investigations such as contrast-enhanced CT/magnetic resonance imaging, endoscopy, and biopsy for early detection and treatment to improve prognosis. The specific expression of the PD-L1 protein and the identification of genomic characteristics may offer new hope for the treatment of small intestine sarcomatoid carcinoma and warrant further research and trials if conditions permit.

## Author contributions

**Data curation:** Xujie Wang, Huan Zhang, Long Li, Jixin Fu.

**Investigation:** Xujie Wang, Huan Zhang.

**Writing – original draft:** Xujie Wang.

**Writing – review & editing:** Xinjian Wang.
